# Diverse Land Use and the Impact on (Irrigation) Water Quality and Need for Measures — A Case Study of a Norwegian River

**DOI:** 10.3390/ijerph120606979

**Published:** 2015-06-17

**Authors:** Gro S. Johannessen, Aina C. Wennberg, Ingrid Nesheim, Ingun Tryland

**Affiliations:** 1 Norwegian Veterinary Institute, P.O. Box 750 Sentrum, NO-0106 Oslo, Norway; 2 Norwegian Institute for Water Research, Gaustadalléen 21, NO-0349 Oslo, Norway; E-Mails: aina.charlotte.wennberg@niva.no (A.C.W.); Ingrid.Nesheim@niva.no (I.N.); Ingun.Tryland@niva.no (I.T.)

**Keywords:** water quality, fecal indicator bacteria, *E. coli*, municipal measures, irrigation water, fecal contamination source

## Abstract

Surface water is used for irrigation of food plants all over the World. Such water can be of variable hygienic quality, and can be contaminated from many different sources. The association of contaminated irrigation water with contamination of fresh produce is well established, and many outbreaks of foodborne disease associated with fresh produce consumption have been reported. The objective of the present study was to summarize the data on fecal indicators and selected bacterial pathogens to assess the level of fecal contamination of a Norwegian river used for irrigation in an area which has a high production level of various types of food commodities. Sources for fecal pollution of the river were identified. Measures implemented to reduce discharges from the wastewater sector and agriculture, and potential measures identified for future implementation are presented and discussed in relation to potential benefits and costs. It is important that the users of the water, independent of intended use, are aware of the hygienic quality and the potential interventions that may be applied. Our results suggest that contamination of surface water is a complex web of many factors and that several measures and interventions on different levels are needed to achieve a sound river and safe irrigation.

## 1. Introduction

Water is essential for the production of food plants and surface water is used for irrigation all over the World. However, such water can be of variable hygienic quality with respect to occurrence of pathogenic microorganisms and indicators of fecal contamination, e.g., depending on source, weather conditions, topology, and proximity to livestock [1,2]. Surface water can be contaminated from many different sources, such as direct fecal contamination from humans (e.g., from combined sewer overflows (CSOs) during heavy rain or inadequate treatment by on-site wastewater treatment plants) and animals (livestock and wild animals), extreme weather events such as flooding or indirectly from run-off from pastures, sewage leakage, *etc.* Outbreaks of infections associated with contaminated drinking water are well known (e.g., [3,4]) and the association of use of contaminated water for irrigation with contamination of fresh produce is also well established [5,6]. This contamination is especially risky if the fresh produce is typically consumed raw, without risk-reducing measures such as heat treatment. Typical examples of vegetable food consumed raw are lettuce and other leafy greens, berries and fruits. There was an outbreak of *E. coli* O157:H7 infections in Sweden associated with lettuce, which was linked to the use of contaminated irrigation water from a river. Investigations indicated that the water was contaminated from a farm keeping cattle upstream of the irrigation water intake [7].

In Norway, surface water and overhead irrigation are commonly used in open field production of fresh produce such as lettuce. According to the national quality assurance system for agriculture (KSL) [8] farmers are required to analyze at least one water sample each season for *E. coli*, to document the hygienic quality of the irrigation water, although no specifications are given as for when to test the water. There are also other aspects included in KSL such as awareness of origin of contamination and protection of the water source.

Within the EU, four directives regulate water quality: (i) The Water Framework Directive (WFD) [9] aimed at ensuring the good ecological status of waters, (ii) The Urban Waste Water Directive [10] which concerns the collection, treatment and discharge of urban waste water, this directive has a direct bearing on the contamination of bacteria in waters, (iii) The Nitrate Directive [11] has a focus on the prevention of nitrate pollution from agriculture in ground and surface waters; and (iv) The Bathing Water Directive (BWD) [12] which requires Members States to monitor bathing water for at least two fecal bacteria parameters and to inform the public about the status of bathing water. However, no European directive focuses on the hygienic quality of irrigation water and no common European standard applies. In Norway, the municipality is the competent pollution control authority for waste water emissions from individual houses and smaller waste water treatment plants (WWTPs) in towns, while the County Governor is the competent authority for emissions from WWTPs in larger towns and cities. A number of laws and regulations adhere to the wastewater sector including, the Pollution act [13], the Pollution Control Regulations [14], the Water Resources Act [15]. The Planning and Building Act [16] regulates, among other things all planning within the water and wastewater sector. There are also rules for the use of fertilizers [17] and a Regulation for the use of organic manure [18] which are relevant for reducing diffuse pollution from agriculture. Fertilizers are commonly distributed on the fields three times during the season: The first time in early spring (April or May), but not on frozen soil; the second time after the first harvest, and the third time after the second harvest. The regulation for the use of organic manure [18] states that manure shall not include *Salmonella* or infective parasite eggs, and the number of thermotolerant coliform bacteria shall be less than 2500 cfu/g dry matter. Products have to be stabilized in order not to cause environmental problems during use and storage. In addition the municipality may issue local regulations if necessary to improve water quality conditions, and the municipality shall adopt sanctions by violation. The Lier municipality Regulations for Water and Sewage Fees [19] give the municipality the authority to levy water and sewage charges.

The municipality of Lier (study area) is situated within a river basin which on Norwegian terms yields a high production of various types of fruits, berries, and vegetables. The Lier River, a river which has been used for fishing, bathing and for irrigation of fields since historic times, runs along the middle of the municipality. Depending on the season, irrigation is normally required for plant production in southern Norway. The harvest season for leafy greens is from May to the end of September, and irrigation is normally applied if required throughout this period. The normal mean daily temperature in the study area varies between 10 °C (May) to 16 °C (July) with a monthly precipitation between 60 mm (May) and 100 mm (September) [20]. Presently, the river basin includes a small urban center (Lierbyen), scattered settlements and livestock and vegetable farmers. The Lier River is characterized as a fecally contaminated river. Several research projects (performed in 2000, 2006 and 2012) provide data about the occurrence of fecal indicator organisms (FIOs—In this study fecal coliforms and *E. coli*) and enteric bacterial pathogens in the river. The Lier municipality has carried out routine monitoring for FIOs since 2004, e.g., in connection with work for fulfilling the requirements in the WFD.

Furthermore, the municipality has implemented a number of measures in the recent decade to improve the water quality in the river. Several more are in the pipeline, while others have been identified for evaluation. The objective of the present study was to summarize the data on fecal indicators and selected enteric bacterial pathogens, to assess the level of fecal contamination in the river and its variation, with focus on the impact of weather conditions before sampling, as well as changes in land use in the catchment area. Measures implemented the recent decades to reduce the discharges from the wastewater sector and agriculture, and potential measures identified for future implementation are presented in this paper and discussed related to potential benefits and costs.

## 2. Experimental Section

### 2.1. Study Area

**Figure 1:**
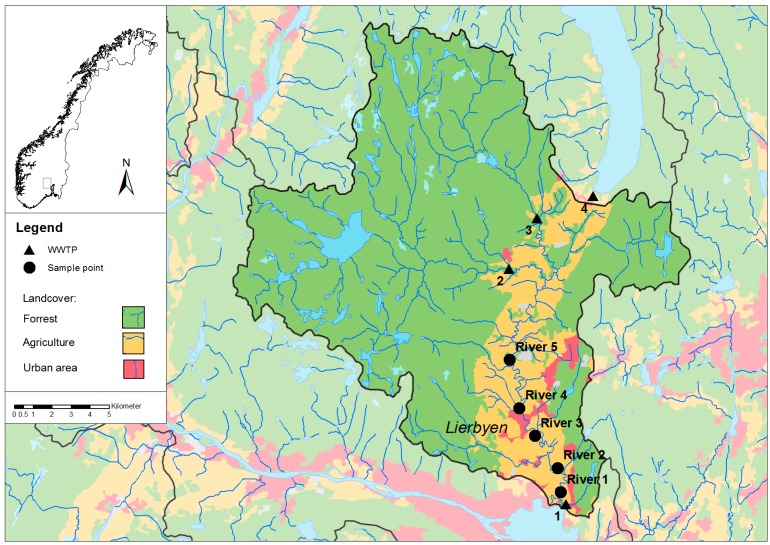
Study area: The Lier River catchment area with different land cover types, the four municipal WWTPs and the municipal river sampling points.

The catchment area of Lier River covers 310 km^2^. It consists of forest, agricultural area (39 km^2^) and some densely populated areas as shown in Figure 1. About 22,000 people live in the catchment area and the agriculture consists of both plant production (cereals, vegetables and fruits) and animal husbandry. A total of 34 irrigation systems using water from the Lier River and tributaries were reported in a public report on water administration from 2012 [21]. The municipality is the second largest producer of vegetables and berries in open fields and the largest greenhouse producer for fresh produce [22].

The Lier River runs in a ravine valley with an outlet to the Drammensfjorden fjord; it is approximately 40 km long and has about 25 tributaries. The water flow in the river is regulated to keep a minimum water flow of 0.7 m^3^/s in the period 15 May to 15 September to protect salmonid fish in the river, and 0.2 m^3^/s for the rest of the year (average flow 2.02 m^3^/s) [23]. Water can be led by a tunnel from a freshwater lake outside the catchment area to maintain the minimum water flow. During warm summer days, when fields are irrigated, the water flow may typically be about 1 m^3^/s (close to this minimum water flow). In periods with heavy rainfalls and snow-melting, the water flow may increase to above 100 m^3^/s and affect certain agricultural areas with flooding.

### 2.2. Water Samples

Since 2004 Lier municipality has collected water samples from the Lier River on pre-set dates approximately once a month during snow and ice free periods at five different places (Figure 1). Until June 2012 the samples were analyzed for fecal coliform bacteria, while from July 2012, samples were analyzed *E. coli* (hereafter FIOs) as an improvement of the routine as *E. coli* is a more reliable indicator than fecal coliforms.

Analysis results have been uploaded to a public database [24], and data from the period 2006 until 2012 were used in this study. The irrigation water samples were collected in three different projects during growth seasons in 2000, 2006 and 2012, all focusing on food safety in primary production of lettuce (and strawberries in 2012). The samples were either collected directly from the water source or from spreaders if in use. Farm A is located upstream river sampling spot 5, farm B is located between river sampling spot 2 and 3, while farms C and D between river sampling spot 1 and 2. The sampling periods and the parameters analyzed are listed in Table 1.

**Table 1:** Sampling periods and parameters analysed for the separate farms.

Farms	Sampling Period	Indicator Bacteria		Pathogens		Total Number of Samples Per Farm Per Year
		Pres. *E. coli* *	*E. coli*	*Campylobacter*	*Salmonella*	
Farm A	19 06–09 08 2000	x	ND **	ND	X why different?	16
	29 05–28 08 2006	ND	x	x	X	19
Farm B	18 06–04 07 2012	ND	x	x	X	4
Farm C	19 06–09 08 2000	x	ND	ND	X	17
Farm D	19 06–09 08 2000	x	ND	ND	X	16
	21 05–24 09 2012	ND	x	x	X	7

* Norwegian Standard 4792:1990. Water analysis—Thermotolerant coliform bacteria and presumptive *E. coli*. Membrane filtration method [25]; ** Not done.

### 2.3. Bacteriological Methods

The samples were analyzed for FIOs using the same methods; for fecal coliforms/presumptive *E. coli* a membrane filtration method was used [25], while *E. coli* was quantified by the MPN method Colilert-18 (IDEXX Laboratories, Wilmington, DE, USA) [26]. The irrigation water was tested for the presence/absence of *Campylobacter* spp. and *Salmonella* by filtering known volumes of water (500 mL in 2006 and 1 liter in 2012) through a 0.45 μm filter (Millipore, Billerica, MA, USA), followed by incubation of the filters using the NMKL methods for *Campylobacter* and *Salmonella* [27,28].

### 2.4. Population, Land Use, and Meteorological Data

Statistics describing the population, land use and agriculture including livestock in Lier municipality were collected from Statistics Norway [29] to describe trends in agriculture and changes in possible bacterial pollution sources in the study period from year 2000 until 2012. Data on grazing land and number of livestock in grazing land were collected from Norwegian forest and landscape institute map AR50 [30]. Document analysis of public reports from the catchment area describing the water quality situation in the river, the various pollution sources from the sewer and waste water treatment system and from agricultural sources have been used for a first evaluation of the fecal bacteria situation in the river [31,32]. These reports also provide information on already implemented, and on planned measures for achieving good ecological status in the river. Other types of data collection techniques include a combination of several informal conversations and semi-structured interviews on the phone, and at meetings in Lier municipality with the Agricultural Office, the Wastewater Treatment Offices and with farmers. Data on precipitation from the Lier weather station was collected from LMT [33] (adjusted for summer time, 24 h values).

### 2.5. Statistical Analysis

The bacterial concentrations were log_10_ transformed before analysis. To look at trends in irrigation water quality in the study period, only results from months with irrigation were included in the analysis. Extra samplings in addition to the monthly sample were excluded from the plot to prevent specific events from affecting/influencing the statistical analysis.

The sampling results for each river sampling site were plotted against sampling date, and a linear regression was performed in MS Excel. A null hypothesis testing using t distribution and n − 2 = 35 degrees of freedom was performed to assess whether there was a significant reduction or increase in fecal bacterial concentrations over time within a 95% confidence interval (*t* score ± 1.690).

A paired two tailed *t*-test was done in excel to compare the water quality in the five different river samples. Results are expressed as average difference in concentrations ±95% confidence interval (CI) calculated based on *t*-distribution in Excel. A two-tailed *t*-test with unequal variances was used to compare the water quality of farm samples to the nearest river sample location.

## 3. Results

In this section we present data from municipal monitoring of the river water, and irrigation water samples collected in three research projects together with measures taken to fulfill the WFD. Since the microbiological pollution is assumed to be of both human and animal origin, expected pollution sources are presented together with measures taken to improve the water quality in the river.

### 3.1. Water Quality Trends in Lier River

**Figure 2:**
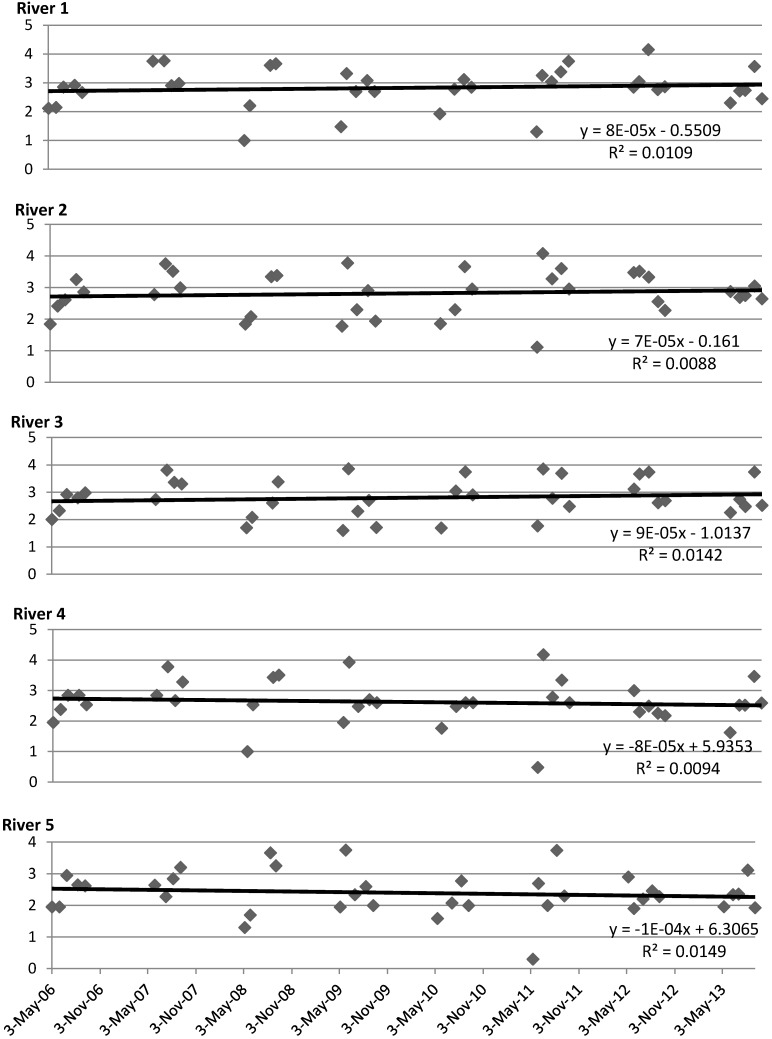
Monthly samples of fecal coliforms or *E. coli* (log_10_ cfu/100 mL) sampled by the municipality in the irrigation period (May to September).

The trends in numbers of fecal indicators in the irrigation period at the different sampling locations are shown in Figure 2 and Table 2. The FIO concentrations were significantly higher in sampling spots downstream of Lierbyen compared to sampling spots upstream of Lierbyen. On average the fecal bacterial concentration increased with 0.53 ± 0.14 log_10_ cfu/100 mL (*p* = 0.003, *N* = 37) from river sampling point 5 to sampling point 4 which is located upstream of Lierbyen, and with 0.35 ± 0.17 log_10_ cfu/100 mL (*p* = 0.045, *N* = 37) from river sampling point 4 (upstream of Lierbyen) to river sampling point 3 located downstream of Lierbyen. There was no significant difference between the three river sampling points downstream of Lierbyen (*p* = 0.73 and 0.91 for river sampling points 3 *vs.* 2 and 2 *vs.* 1, respectively). The general trend from 2006 until 2013 also split between sampling spots upstream of Lierbyen and downstream of Lierbyen with a slightly decreasing bacterial concentration (significant only for river sampling point 4) and a slight increase in bacterial concentrations (significant only for river sampling point 2). The overall trend is the same when excluding samples taken on days where the farmers are less likely to use irrigation water (minimum 10 mm rainfall during the last 3 days or minimum 5 mm rainfall during the last 24 h), see Table 3 (20 samples). However, the trends are a little stronger and the decreasing concentration trend is significant also for river sample 5. The average concentration of all samples is lower for the dry weather samples compared to all samples. For all sampling locations there was a large variation in the FIO concentrations measured, reflecting fluctuations in water flow, weather conditions and pollution sources and sizes.

**Figure 3:**
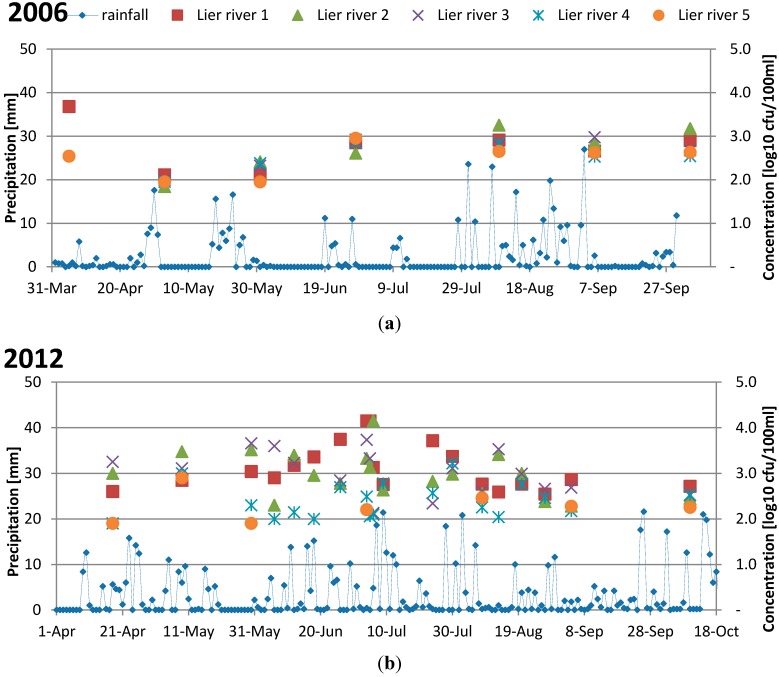
(**a**) daily rainfall (mm) and fecal coliforms (log_10_ cfu/100 mL) in municipal samples from 2006 and (**b**) daily rainfall and fecal coliforms or *E. coli* (log_10_ cfu/100 mL) from 2012.

The daily rainfall and sampling results for two of the study years 2006 and 2012 are presented in Figure 3. No data existed from 2000. A larger variation in concentrations of FIOs in the samples can be observed in 2012 compared to 2006 when the levels were less variable. The season 2012 was characterized by more frequent rainfall compared to 2006 which was warm and dry.

**Table 2:** Average and variance of fecal bacterial concentrations (log_10_ cfu/100 mL) in river samples 1 to 5 with linear regression analysis (Y = ax + b) and result of null hypothesis based on *t*-distribution (37 samples analyzed for each river sampling spot).

Samples	Average	Variance	a	b	R^2^	R	*t* obs	Significant *t* = ±1.69
River 1	2.83	1.0–4.2	8 × 10^−5^	−0.55	0.0109	0.10	0.58	no
River 2	2.82	1.1–4.1	7 × 10^−5^	−0.16	0.0088	0.09	1.96	yes
River 3	2.80	1.6–3.9	9 × 10^−5^	−1.01	0.0142	0.12	1.55	no
River 4	2.62	0.5–4.2	−8 × 10^−5^	5.94	0.0094	0.10	−1.80	yes
River 5	2.40	0.3–3.7	−1 × 10^−4^	6.31	0.0149	0.12	−1.35	no

**Table 3:** Average and variance of fecal bacterial concentrations (log_10_ cfu/100 mL) in only dry weather river samples 1 to 5 with linear regression analysis (Y = ax + b) and result of null hypothesis based on *t*-distribution (20 samples analyzed for each river sampling spot).

Samples	Average	Variance	a	b	R^2^	R	*t* obs	Significant *t* = ±1.734
River 1	2.5	1.0–4.2	3 × 10^−3^	−8.39	0.101	0.317	0.094	No
River 2	2.47	1.1–3.5	1 × 10^−4^	2.25	0.022	0.148	11.160	Yes
River 3	2.54	1.6–3.7	2 × 10^−4^	−4.58	0.047	0.217	1.616	No
River 4	2.26	0.5–3.3	−5 × 10^−5^	4.19	0.004	0.065	−3.537	Yes
River 5	2.03	0.3–3.2	−2 × 10^−4^	8.11	0.046	0.213	−2.940	Yes

### 3.2. Results from Analysis of Irrigation Water

**Figure 4:**
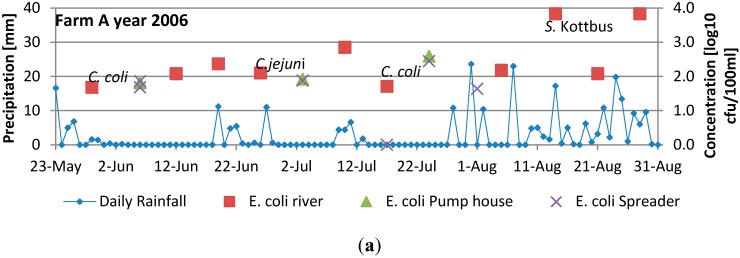

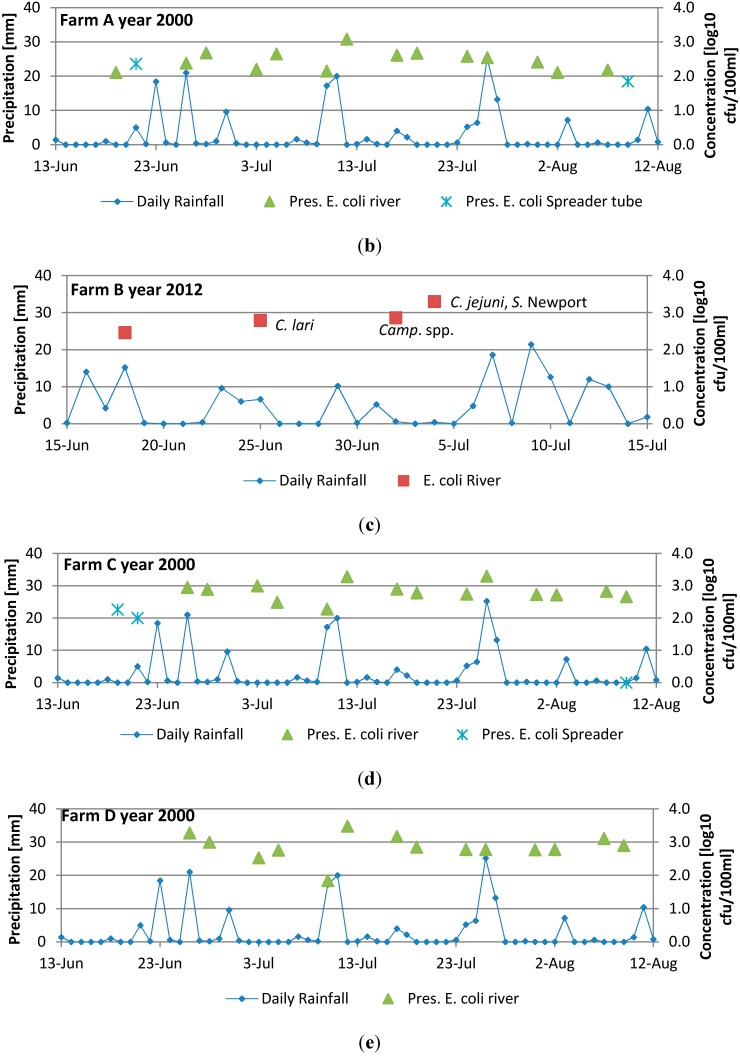

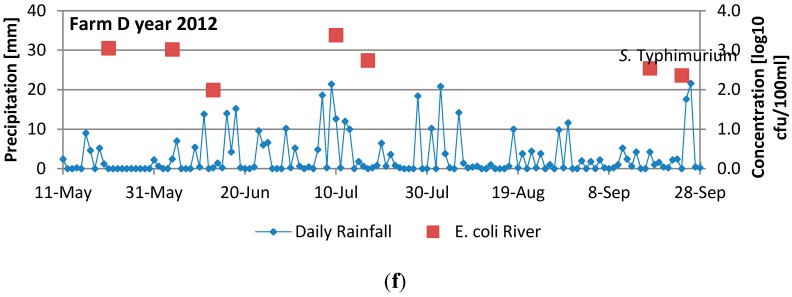
Daily rainfall, concentration of FIOs (log_10_ cfu/100 mL) and presence of pathogens during the harvest season. When a pathogen is plotted in the figure, this signifies the isolation of the pathogen on the sampling date. (**a**) daily rainfall and presumptive *E. coli* in 2000 at Farm A, (**b**) daily rainfall, *E. coli*, *Campylobacter* and *Salmonella* in 2006 at Farm A, (**c**) daily rainfall, *E. coli*, *Campylobacter* and *Salmonella* in 2012 at Farm B, (**d**) daily rainfall and presumptive *E. coli* in 2000 at Farm C, (**e**) daily rainfall and presumptive *E. coli* in 2000 at Farm D, (**f**) daily rainfall, *E. coli* and *Salmonella* in 2012 at Farm D.

The results from the individual farms are presented in Figure 4 [34,35]. The results indicate a large variation of the numbers of indicator bacteria in the water, ranging from below 0 log_10_ cfu/100 mL to more than 3.83 log_10_ cfu/100 mL. However, it can be observed that most of the water samples have levels of indicator bacteria between approximately 2 and 3.5 log_10_ cfu (MPN)/100 mL. When comparing results from the analysis of irrigation water samples collected in the source (*i.e.*, the river) with the results from the closest municipal sampling point, no statistically significant difference could be observed (2006: Farm A: *p*: 0.90) (2012: Farm B: *p* 0.58 and 0.32 and D: *p* 0.23 and 0.36).

Pathogenic bacteria were also occasionally isolated from the water, with *Campylobacter spp*. Being isolated from six samples (three in 2006 and three in 2012 ) and *Salmonella* from three samples (one in 2006 and two in 2012) [35]. In some cases, peaks in numbers of fecal indicators follow directly after heavy rainfall, but this is no always the case. There are also no obvious association between presence of pathogens and rainfall. For example for farm B in 2012, both *C. jejuni* and *Salmonella* Newport were isolated after a period with no precipitation.

### 3.3. Trends in Land Use

#### 3.3.1. Pollution Source: Farm Animals—Grazing Lands

The number of farms with livestock and the number of livestock present on farms during winter is shown in Table 4. Fecal excreta from animals in stables and barns are stored in tanks and used as fertilizers on the fields (in fall and spring) in Lier. Tanks are closed to prevent leakage to local waters/rivers. During the summer season animals are allowed out in pastures which might be close to the rivers. Dairy cattle is mostly kept on pastures close to the farm, but sheep and some cattle are transported to large grazing lands in the forest for the summer season after lambing. Within the catchment area there is a total of 156 km^2^ of grazing land with approximately 4000 sheep and 460 cattle [30]. Horses may also contribute to diffuse sources of fecal contamination. There are about 100 horses in the area and a few large and many small stables.

**Table 4:** Population, agriculture and farm animals in Lier municipality in year 2000, 2006 and 2012 (based on numbers from Statistics Norway [29]).

	2000	2006	2012
Population Lier municipality	21,308	22,700	24,177
Urban areas	16,395	17,295	19,190
Number of farms	273	198	164
Number of farms with vegetable production	58	48	31
Number of farms with animals	98	78	71
Cattle (total)(winter)	2006	2126	1841
Cattle dairy production	460	412	310
Sheep (Winter)	2147	2722	3055
Chickens	7367	5728	0

**Figure 5:**
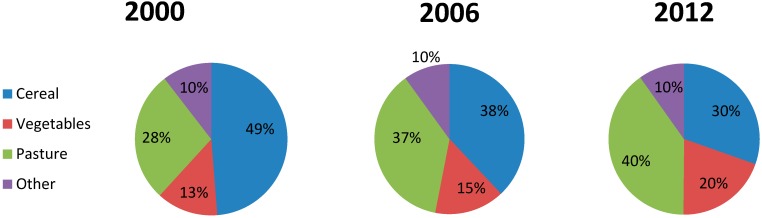
Use of agriculture area in Lier municipality in year 2000, 2006 and 2012 (based on numbers from Statistics Norway [29]).

There has been a shift in agriculture to fewer and larger farms. The total area used for agriculture is almost the same; 37.6 km^2^ in 2000 and 36.7 km^2^ in 2012 [29]. There has also been a shift from cultivation of cereal towards vegetables and pastures, see Figure 5.

#### 3.3.2. Pollution Source: Municipal

Waste water is handled by either four WWTPs (See Figure 1) managed by the municipality (~80% of population) or by private sanitation systems for separate households in scattered settlements (~20% of population). Except for one WWTP (WWTP 2 in Figure 1) for approximately 560 inhabitant equivalents (IE) with effluent to a tributary of the Lier River, the other WWTPs do not discharge to the river. However, leakages and overflows from the transport system (e.g., emergency overflows from pump stations, combined sewer overflows) may contribute to the fecal load, especially since much of the 250 km of pipelines are in ditches close to the riverbed. The smallest WWTP (approx. 100 IE) (WWTP 3 in Figure 1) is basically a sludge separator where the sludge is emptied by a tanker while the effluent is discharged to the river. Water analysis downstream of the sludge separator shows little change in the concentration of FIOs relative to upstream this WWTP (fecal coliforms/*E. coli* in the range 2 to 600 cfu/100 mL, average 250 cfu/100 mL). The small private sanitations systems are sludge separators, holding tanks, onsite wastewater treatment plants or latrines [31]. Sludge separators will retain particulate matter, but little of the bacteria are removed before the water is infiltrated in the ground (average 40%–50% removal) [36]. In theory, infiltration could be a good method for pathogen removal (average 99.99% removal), however, the soil in Lier consists of much clay with insufficient infiltration. On-site WWTPs can have several methods of treatment (chemical and/or biological), and some include a disinfection step that can reduce the number of bacteria significantly (90%–99% removal) under ideal situations [36].

### 3.4. Measures Taken to Improve Water Quality in the River

Lier municipality has implemented a number of measures in the catchment in line with its objective of good ecological status of water bodies within 2015 (WFD) and in line with the objective of ensuring that water from the river can be suited for irrigation of fresh produce. Two inter-municipal offices are responsible for ensuring that the wastewater systems have minimum leakage and discharge to the Lier River, and hence responsible for implementing necessary measures. An inter-municipal agricultural office is responsible for measures to reduce point source and diffuse pollution from agricultural sector. A number of measures have been implemented by the wastewater sector the last 10–15 years; (see Table 5) and an important emphasis has been on reducing effluents from combined sewage overflows (CSOs) by installing separate pipes for storm water run-off and sewage, an objective which will be completed in 2015. Another important measure has been to modernize the sewer pump stations by installing alarm systems to signal leakage or other discontinuations of the system. To provide for these measures the municipal sewage sector has had budget of more than five million US $ annually the last five to ten year. A current focus is to replace WWTP 3 (Figure 1) with a new WWTP including biochemical treatment; it has not yet been decided whether this new plant will have a hygienic treatment step. Replacement of old pipes is an ongoing action which will continue systematically in the catchment. Connecting non-sewered areas to the municipal centralized sewage system has been identified in the Lier action plan for sewage as the next main focus areas, parallel with ensuring satisfactorily treatment by on-site WWTPs in those areas which will continue to have decentralized wastewater management [32]. These latter measures are of a different character as they involve private action and expenditure.

The main remediation measures to reduce pollution implemented by the agricultural sector have been related to the objective of reducing runoff of phosphorus into rivers. Fencing as a measurement for keeping livestock away from streams and the river has been discussed, but this has not been implemented as it has been considered inconvenient in the area [37] (see Table 5). Table 5 presents a number of potential measures identified during discussions with the agricultural office and with farmers in the area related to the overall objective of keeping livestock away from the river during the irrigation season. Another potential source of bacteria comes from manure where an important measure would be inspection of farmer practices to ensure that rules and regulation for how to store and apply manure are followed (Table 5).

**Table 5:** Identified risks and measures for pollution sources.

Situation	Risk-assessment	Identified Measure *	Benefits/Feasibility	Costs
**WWTP** 2 biochemical treatment: Sewage from 560 IE.	Sludge brought to WWTP 1 for hygienic treatment. Low risk	Plans have been adopted for adding a dewatering component to the treatment plant.	High benefits of having a WWTP in the municipality.	High costs for Lier Municipality
**WWTP** 3 sludge separator: Sewage from 100 IE.	High risk of effluents with bacteria through discharge of waste water	A new WWTP 3 is planned, treatment steps will include biochemical treatment.	High benefits of having a modern effective treatment plant.	High cost for Lier Municipality.
Pipes from private houses are the owners’ responsibility	Risk of leakage, level of discharge depends on location of leakage.	Frequent monitoring. Replace all old pipes and pipes with poor quality.	Will contribute to the reduction of *E. coli*.	Costs will be on private owners. Costs relatively high.
Leakage from pumping stations	Reduced risk due to the alarm systems to signal leakage.	*Alarm systems have been installed, and pump stations have been modernized*. Shorter action time can be implemented (presently 8 h).	Will contribute to the reduction of *E. coli*.	Relatively high costs
Leakage from old/poor quality pipes. Combined sewage overflow (CSO)	Presently reduced risk as most old pipes have been replaced. Increased risk with rain and heavy rain.	Replacement of old municipal pipes. *All CSO should be replaced with a separate system by 2015 according to the municipality plan*.	A modern sewerage system reduces *E coli*.	High cost for the municipality. More than 5 M US $ annually
**Private Sewage system:** Discharge of waste water from 700 sludge separators (SS) mechanical treatment. Septic tanks are emptied by the municipality.	Discharge of *E. coli* depends on conditions for infiltration in the ground/soil. Ground/soil conditions in Lier are generally not suited; Risk of leakage from septic tanks; Risk depends on distance from SS to stream/river.	*All sludge separators and ground conditions are mapped. A certified company for emptying septic tanks. Unnoticed controls. A fine if rules broken.*	Measures will reduce bacteria levels, but discharge can still be high.	Costs are taken by the municipality, works tasks are regulated by the Pollution Act.
		Emptying septic tanks more frequently.	Important benefits if tanks leak.	Costs are on private households, experienced costs vary.
		Add treatment steps to SS, organic, chemical and or hygienic treatment.	All treatment steps highly beneficial. Supervision of several systems needed	Intermediate direct cost level, but high costs on monitoring of the different systems.
		Replace old SS with on-site biochemical and hygienic treatment plants	Assuming good supervision, this action will greatly reduce *E. coli* levels.	Experienced costs vary among households, costs about $16,500 per plant
		Connect the private sewage systems to centralized sewage system.	Removal of local discharge of *E. coli* to streams.	Relatively high private direct costs. Municipality cost of installing pipes.
On-site biological WWTP (removes 60% *E. coli*) (2 in the basin);	2/3 of the private treatment plants were established before the Pollution Control regulation, these have unacceptable discharge to rivers and streams;	*Requirements for operating supervision of treatment plants. According to the municipal documents: Those with unacceptable treatment will have to either: 1. Add an extra cleansing step, or 2. Replace the plant with a new treatment plant, or 3. connect to centralized system. Building permits for new houses requires that sewage is connected to centralized sewage, or satisfactorily treatment of on-site sewage*	Private treatment plants which includes all treatment steps, with an authorized supervision has acceptable treatment.	Treatment plants with several different add-on-systems will require several different supervising agreements which can be costly.
On-site chemical WWTP (removes 99% *E. coli*) (5 in the basin); On-site biochemical WWTP (99% *E. coli*) 51 in basin);	Risk of high effluents to the river dependent on distance to stream/river;			
On-site with hygienic treatment WWTP (20 in the basin)	Risk dependent on supervision and management of private plants.			
Animals grazing, trampling and depositing feces nearby, or in the stream/river.	Significant risks where animals can go down to river/stream to drink and deposit faeces; Increased risk during rainfall; Risk level reduced with long distance to river and to area for water abstraction; Risk level dependent on the ratio, animals and river water flow.	Fencing stretch of river	Potentially a significant benefit for reducing fecal bacteria levels [38].	Costs of setting up the fence, and of providing alternative drinking source for livestock. Relatively high costs.
		Provide drinking water for animals away from the stream	If placed in the right place, this will reduce deposits of faeces along streams.	Costs refer to that of providing an alternative water source sufficiently away from the river. Mainly onetime cost, comparatively low.
		Place salt stone away from stream and river	Some impact.	Low
		Provide fodder away from stream and river		Low
Horse riding along streams. Around 100 horses in Lier	Horse excreta contribute to *E. coli* in the river.	Facilitate for horse tracks away from stream and rivers.	Will reduce the contribution of *E. coli* to the river.	Cost is comparatively low
Run off from barns and stables	Risk depends on distance to stream/river.	Sufficient and closed storage	High	Capital cost to ensuring satisfactorily storage
Runoff from fertilized fields to stream/river; Emptying fertilizer containers	Risk depends on distance to streams/river, and practices of applying manure.	Vegetated buffer strips, Pond systems; Applying manure in dry weather, and avoid irrigation periods	Relatively high benefits [39].	The main cost refers to the “loss” of land area for reforestation.

* Identified measures marked in italic have already been implemented.

## 4. Discussion

Land use patterns have a significant impact on the quality and quantity of water resources by means of changing land cover, impact on river discharge levels, and by increasing polluted effluents to waters. Furthermore the relationship between land and water use is mutually dependent as the changing characteristics of one can impact the potentials for use of the other. It is this relationship which inspired the development of the many “integrated approaches”, e.g., the WFD, Integrated Water Resource Management (IWRM) *etc.* aiming for holistic management of land and water. Lier municipality is characterized by diverse land use interests, a river transcending through a rural, semi-urban and urban landscape providing both drinking water for livestock and irrigation water for vegetable production. The river, however, suffers from fecal pollution from private and municipal waste water systems, and from diffuse and point sources from the agricultural sector. These land use practices contribute to the high FIO level in the river, making it less suitable as irrigation water. This land use situation represents a typical example where collaboration and coordinated governance between the municipal agricultural, environmental, and waste water offices are needed to identify targeted measures for reducing FIO levels in the water. Below we discuss this situation, and the various proposed measures to reduce contamination of the river related to their feasibility and their effectivity.

### 4.1. Water Results

The results from the irrigation water samples and municipal samples that the levels of FIOs at the different sampling points are in the same area, although not directly comparable. With FIO numbers varying between log_10_ 2 and log_10_ 4 cfu/100 mL and occasional isolation of pathogens such as *Salmonella* and *Campylobacter*, our results are comparable to other results collected in the same context elsewhere [40,41,42,43]. In Holvoet *et al.* [41] 59% of the water samples harbored *E. coli* in numbers varying from log_10_ 0.0 to 3.6 cfu/100 mL with a median of log_10_ 1.5. They also isolated VTEC, *Salmonella* and *Campylobacter* from the samples. Pagadala *et al.* [43] also found that source of irrigation water was a significant factor for FIO levels, where ground water had lower levels than surface water. The sources of surface water were described as ponds, creeks and streams. Strawn *et al.* [40] studied how landscape and meteorological factors affected the prevalence of foodborne pathogens in fruit and vegetable farms in US. They found that factors related to water, temperature, proximity to different land covers and precipitation influenced the detection of *L. monocytogenes* and *Salmonella*.

It is, however, not the single results that are interesting, as these only gives a snapshot of the situation when the sample was collected, but the trends in numbers. In the present study we observed a slight increase in FIOs in the samples collected by the municipality in the samples downstream off the most densely populated area (Lierbyen), while there was a slight decrease of numbers in the samples collected upstream of Lierbyen. There has however been a 10% increase in population in Lier during the last 6 years, and that increase has occurred in the urban areas such as Lierbyen with a consequent increase of pressure on the sewage system.

Since the river is used as an irrigation water source for produce, such as lettuce and strawberries, it is important to consider the consequences of using this water. Studies carried out to survey products and risk factors during primary production suggest that contaminated irrigation water is major source of contamination of the lettuce [5,41]. However, results from Norway indicate that although there is a continuous background contamination, the occurrence of FIOs and pathogens on the products at harvest are rather low [35,44]. With respect to water sampling, vegetable producers in Norway are required to take at least one irrigation water sample a year [8], but distributors of fruits and vegetables often request that more than one sample is tested and also have requirements for when the samples are collected. However, as discussed above, one single sample gives a snapshot of the situation at the time of sampling. In order to get a better result of the actual irrigation water quality, the samples should be collected from the irrigation point during irrigation, *i.e.*, from the spreaders themselves. Interestingly, a study from New York State, USA revealed that only 27% of growers reporting using surface water and overhead irrigation tested the water [45]. This suggests that there should be a focus on irrigation water quality for all vegetable producers.

### 4.2. Measures Identified to Improve the Water Quality

Measures can be categorized based on whether these demand action by the municipality sewage sector, or if actions should be undertaken by society, that is private households and farms. For the latter, costs, that is capital expenditure and resource use, have to be taken by the private actors while benefits of remediation actions to a large extent goes to the community at large and the vegetable producers specifically.

#### 4.2.1. The Wastewater Sector

The situation in the Lier municipality, where the wastewater treatment plants and the sewerage infrastructure are updated for better treatment, reflects the situation elsewhere in Europe [46,47]. These are actions that have gained increased emphasis the last decade along with implementation of the WFD and the BWD. Despite this, it can be argued that it will take at least one more decade before old pipes have been replaced, CSOs separated, and technically backward treatment plants modernized. Furthermore, even a top modernized system will from time to time experience leakage, due to incorrectly connected pipes or other incidences. It may be unrealistic to expect that this system can be entirely closed. Hence an important measure would be improvement of the surveillance and alarm systems for the waste water sector and decrease the response time when an incidence happens. Effective sampling procedures to detect too high bacteria levels during the irrigation season, alongside continuous remediation actions to improve irrigation water quality are needed.

The WWTPs in Lier are currently being upgraded and a new plant constructed to achieve satisfactorily treatment of waste water (biochemical & hygienic treatment), hence it can be regarded as ineffective to direct further measures at this system [32]. Furthermore, the combined sewerage system has now largely been replaced by a separate system. The current priority of the municipality is a focus on reducing discharges from decentralized sewerage, *i.e.*, households with septic tanks and poor waste water treatment conditions. The aim during the next decade is to ensure satisfactorily treatment of sewage from the 700 households which currently have sludge separators, either by connecting to municipality sewers or by hygienic treatment in on-site WWTPs. This target however, can be considered more complicated as expanding sewer infrastructure demands collaboration and coordinated actions among different municipality sectors which can be time consuming. The municipality sectors such as the agricultural office and the sewerage sector, and the planning sector as a general situation often have different priorities and focus areas. Furthermore, not all households may be interested in, or have economic capacity to connect to the municipal sewerage. Financial support is available, but the main part has to be spent by the household. In order to pursue households to spend the needed capital, information campaigns, coordinated actions in the area, and laws to support the municipal decision are important [48]. Clearly increased awareness and knowledge about the situation of contaminated irrigation water and the risk for the producer and the consumer are important for efforts aiming at reduced FIO levels in the river. As the major part of the 700 household have very dissatisfactory sewage treatment, ensuring effective treatment of sewage from these houses should reduce discharge to the river, however among these, priority should be placed on those in the neighborhood of rivers. Source tracking methods targeting human fecal indicators can help indicating which areas should be prioritized first.

#### 4.2.2. Agriculture Sector

Several papers have documented significant sources of FIOs to rivers originating from agricultural practices. The FIOs enter the river through soil leaching and surface run off from cattle manure spread on cultivated areas, but also through direct deposits from wild life animals and birds, and grazing livestock [46,49,50,51]. In Lier, FIOs from wild animals and birds are comparatively low, but the relative high number of grazing animals in the proximity of streams in early spring is a likely source of FIOs, as are run-off from other agricultural activities. Several measures to reduce FIOs derived from farms and livestock can be implemented (Table 4), however, since for the farmer most measures are costly and impractical, the focus should be directed at particularly harmful practices, and measures should be evaluated for their effectivity and for their convenience. Trampling livestock in streams in the relative neighborhood (1–3 m) of the river during the irrigation season should be avoided [38]. However, not all area may be suitable for fencing, as the proximity to rivers may be very steep, or soil very instable providing fencing to be difficult. An alternative which can be considered would be to provide drinking facilities away from the stream to attract livestock and thereby reduce their presence alongside streams. Consciousness about where to provide fodder, salt stones or shelter areas for livestock to avoid creating pollution hotspots for run-off to streams and rivers is needed. Such measures are normally not cost demanding, but a matter of being conscious of the impact of actions. It is well recognized that applying organic fertilizers to fields contribute to FIO in run-offs [50,52]. Strict control of storage of manure, and of timing for applying manure should be ensured. Preferably, manure should not be applied during irrigation season if this implies run-off to streams and rivers. However, common for all these issues is the need to increase the awareness about these issues among livestock farmers and that of society in general.

## 5. Conclusions

The results presented here indicate that despite the various measures which have been implemented to improve water quality in the Lier River; the numbers of FIOs in the lower part of the Lier River have not been reduced. This does not mean that the measures taken to reduce contamination have been without effect, but that the increased population and change in land use practices have increased the inputs of FIOs to the river more than the measures have resulted in decreased numbers. Improving water quality is a continuous process and changes in practices, land use and population pattern can affect the contamination/pollution situation of the river. Our results suggest that contamination of surface water, such as the river described here is a complex web of many factors and that several measures and interventions on several levels (in particular by the municipal wastewater and the agricultural sector) are needed to achieve a sound river and safe irrigation. This calls for increased collaborative and coordinated governance among sectors which are based on awareness of the importance of safe irrigation water, firstly for public health, but also to support the multi-functional economic activities in the catchment. More emphasis should be placed on awareness regarding hygienic quality and the potential interventions which farmers can do, and practices which should be avoided, such as for example placing fodder stations close to the river. As part a holistic and effective management of land and water interactions are surely also interventions for vegetable farmers to ensure safe irrigation (e.g., drip irrigation, stop irrigation for a period prior to harvest *etc*.), however the overall goal is that of achieving a sound river.
